# Design of clinical cardioprotection trials using CMR: impact of myocardial salvage index and a narrow inclusion window on sample size

**DOI:** 10.1186/1532-429X-17-S1-P90

**Published:** 2015-02-03

**Authors:** Henrik Engblom, Einar Heiberg, Svend Eggert Jensen, Jan Erik Nordrehaug, Jean-Luc Dubois-Randé, Sigrun Halvorsen, Sasha Koul, David Erlinge, Dan Atar, Marcus Carlsson, Håkan Arheden

**Affiliations:** Cardiac MR group Lund, Dept. of Clinical Physiology, Lund University, Lund, Sweden; Department of Cardiology, Aalborg University Hospital, Aalborg, Denmark; Department of Cardiology, Haukeland University Hospital, Bergen, Norway; Department of Cardiology, Henri Mondor Hospital, Creteil, France; Department of Cardiology B, Oslo University Hospital Ullevål, and Faculty of Medicine, University of Oslo, Oslo, Norway; Department of Cardiology, Lund University Hospital and Lund University, Lund, Sweden

## Background

Cardiac magnetic resonance imaging (CMR) can be used to determine both myocardial infarct (MI) size and myocardium at risk (MaR), enabling assessment of myocardial salvage index (MSI). MI size as assessed by hyperenhancement on late gadolinium enhancement (LGE) has been shown to decrease approximately 25% during the first week after infarction. The aim of this study was to determine to what extent assessment of MSI and a narrow inclusion window affect the number of patients needed to reach sufficient statistical power in a clinical CMR cardioprotection trial.

## Methods

Control subjects (n=91) from the recent CHILL-MI^1^ and MITOCARE^2^ cardioprotection trials, examined by CMR 2-6 days after acute reperfusion therapy, were used to assess the difference in sample size required to reach sufficient statistical power when using MI size alone compared to MSI as outcome variable. In addition, 22 patients undergoing CMR at day 1 and 7 after acute reperfused infarction from a previous follow-up study^3^ were included to assess to what extent sample size is affected by the decrease in hyperenhancement seen during the first week after infarction. The variability of MI size by LGE, MaR by contrast-enhanced SSFP and MSI was used to simulate 100.000 clinical trials for different assumed treatment effects to determine the number of patients needed to reach sufficient statistical power.

## Results

**Figure 1:**
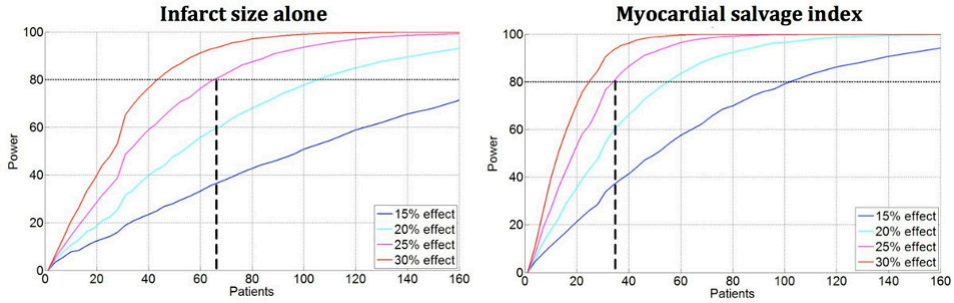
The number of patients needed in each arm to reach sufficient statistical power.

**Figure 2:**
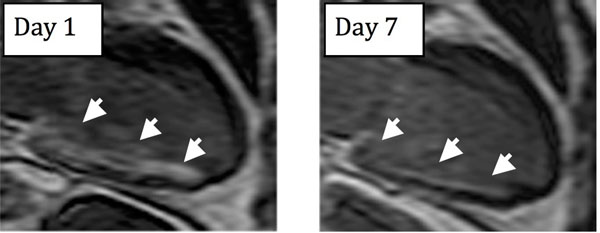
Change in hyperenhancement (arrows) day 1 and 7 after acute reperfusion therapy in a patient with inferior infarction.

For an assumed effect of 25 % reduction in MI size by a cardioprotection treatment, the number of patients needed to reach sufficient statistical power can be reduced by 48% (34 patients in each arm versus 65) if using MSI instead of MI size alone as outcome variable (Figure 1). If a fixed time point for the CMR examination is used instead of an inclusion window of 1-7 days after infarction, the number of patients needed to reach sufficient statistical power decreased by 31% for infarct size alone and 23% for MSI. Figure 2 shows an example of the reduction of hyperenhancement between day 1 and 7.

## Conclusions

There is a significant reduction in sample size needed to reach sufficient statistical power in CMR cardioprotection trials when using MSI instead of MI size alone as outcome variable. In addition, sample size can be further reduced by narrowing the inclusion window during the first week after the acute event.

## References

[1] Erlinge (2014). JACC.

[2] Atar (2014). EHJ.

[3] Engblom (2009). Circ CI.

